# Roles of Seed and Establishment Limitation in Determining Patterns of Afrotropical Tree Recruitment

**DOI:** 10.1371/journal.pone.0063330

**Published:** 2013-05-14

**Authors:** Connie J. Clark, John R. Poulsen, Doug J. Levey

**Affiliations:** 1 Nicholas School of the Environment, Duke University, Durham, North Carolina, United States of America; 2 Department of Biology, University of Florida, Gainesville, Florida, United States of America; 3 National Science Foundation, Arlington, Virginia, United States of America; University of Marburg, Germany

## Abstract

Quantifying the relative importance of the multiple processes that limit recruitment may hold the key to understanding tropical tree diversity. Here we couple theoretical models with a large-scale, multi-species seed-sowing experiment to assess the degree to which seed and establishment limitation shape patterns of tropical tree seedling recruitment in a central African forest. Of five randomly selected species (*Pancovia laurentii*, *Staudtia kamerunensis*, *Manilkara mabokeensis*, *Myrianthus arboreas*, and *Entandophragma utile*), seedling establishment and survival were low (means of 16% and 6% at 3 and 24 months, respectively), and seedling density increased with seed augmentation. Seedling recruitment was best explained by species identity and the interaction of site-by-species, suggesting recruitment probabilities vary among species and sites, and supporting the role of niche-based mechanisms. Although seed augmentation enhanced initial seedling density, environmental filtering and post-establishment mortality strongly limited seedling recruitment. The relative importance of seed and establishment limitation changed with seed and seedling density and through time. The arrival of seeds most strongly affected local recruitment when seeds were nearly absent from a site (∼ 1 seed m^2^), but was also important when seeds arrived in extremely high densities, overwhelming niche-based mortality factors. The strength of seed limitation and density-independent mortality decreased significantly over time, while density-dependent mortality showed the opposite trend. The varying strengths of seed and establishment limitation as a function of juvenile density and time emphasize the need to evaluate their roles through later stages of a tree’s life cycle.

## Introduction

A fundamental challenge of community ecology is to determine the processes that govern patterns of species diversity and composition. In highly diverse communities like tropical forests, recruitment limitation can facilitate species coexistence when superior competitors fail to have any viable juveniles at otherwise suitable sites, thus slowing the exclusion of inferior competitors from the community by allowing them to win sites by forfeit [1], [2], [3]. Recruitment limitation can result from multiple processes occurring at various life history stages, including seed production, dispersal and viability, competition for space and resources, predation, and herbivory [4]: although recruitment cannot occur without seed arrival, seed arrival is no guarantee of recruitment. Recruitment limitation, therefore, needs to be assessed through the lens of both dispersal and post-dispersal processes [5]. What then is the relative importance of dispersal versus post-dispersal processes, such as environmental filtering and niche differentiation, in structuring tropical forests at a given place or in a region?

Individuals are both most abundant and vulnerable to death at the beginning stages of recruitment (*i.e.*, the transition from seed to seedling), and thus processes operating early in the plant life cycle may disproportionately influence the structure, dynamics, and species composition of communities [6], [7]. Two processes, (1) seed and (2) establishment limitation, are thought to largely explain how limitations to recruitment of seedlings influence overall community structure and diversity [5], [8], [9], [10], [11]. Seed limitation is defined as the failure of seeds to arrive in saturating densities at all potential recruitment sites because of low population-level seed production and/or a lack of dispersal to available sites. Establishment limitation is defined as the lack of suitable microsites for recruitment, given the arrival of a sufficient density of seeds. Establishment limitation can be partitioned into several processes or stages that occur between seed deposition and recruitment into the adult population [12].

Both seed and establishment limitation have been shown to occur in natural systems [11], [13]. When seed limitation dominates, the abundance and distribution of species is driven by dispersal, and community assembly can be viewed as a lottery system, where sites are “won” based solely on arriving seed densities [14], [15], [16]. When establishment limitation dominates and seedling recruitment varies across sites and among species, species abundance and distribution are determined by the functional traits and competitive ability of species, regeneration niches, and the relative abundance and quality of appropriate microsites [8], [11], [17], [18]. In the first case, dispersal events maintain unlimited numbers of species as long as the traits that render a species more or less seed-limited trade off with competitive ability [3], [19]. In the second case, post-dispersal niche-based processes underlie community diversity. Some studies interpret seed limitation as evidence that stochastic dispersal processes drive community diversity [20], as it can be perceived as a random process by which propagules arrive at new locations and thus contribute to ecological drift [16]. While we distinguish between dispersal and niche-based processes for convenience sake, we take the view that dispersal is not a strictly stochastic, neutral, or niche-free process, as it differs among species (e.g. [11]) and evolves by natural selection [21].

The degree to which populations are limited by seed dispersal/limitation relative to establishment limitation can be quantified with seed addition experiments, whereby seeds are added to sites (thus decreasing or removing the magnitude of local seed limitation) and seedling recruitment is then compared to control plots without added seeds. When seedling growth and survival in experimental plots is followed over time, seed addition experiments can further isolate the specific mechanisms of establishment limitation. In particular, decoupling density-independent and density-dependent mechanisms is important for understanding tropical tree diversity because some of the best-supported models of species diversity invoke density-dependent mortality [22], [23], [24], [25], [26], [27]. Density-dependent mortality is thought to constrain locally abundant species, which opens space for otherwise less successful species, thus promoting co-existence by creating a rare-species advantage.

To assess the relative importance of dispersal and post-dispersal, niche-based, processes, we initiated a large-scale seed addition experiment to empirically quantify the degree to which seed and establishment limitation drive tropical tree recruitment. In a mixed tropical forest in the Congo Basin, we sowed ∼40,000 seeds of five randomly-selected trees species in stations across >300,000 ha of heterogeneous forest. At each station we augmented seeds of each species at seven densities, ranging from 0 to 2000 times average ambient seed rain densities, and monitored seedling recruitment for two years. We coupled our experiment with models of seedling recruitment (realized and fundamental limitation) to evaluate the relative importance of seed and establishment limitation in the recruitment of tropical tree seedlings. For realized seed limitation, we estimated a single plot-level effect size for each species and seed augmentation combination. We then assessed the conditions (spatial and temporal variation, seed and seedling density, and adult tree density) that affected the relative importance of seed and establishment limitation. For fundamental limitation, we fitted the data to models of seedling recruitment, and then we used the parameter estimates to quantify seed limitation and to decompose establishment limitation into density-independent and density-dependent mortality. We then compared the strength of these processes in relation to seed density and through time. For both realized and fundamental, we parameterized the models for individual species and all five species combined, but we emphasize the community level effects in this paper.

## Methods

### Study Area

This study was conducted in the north of the Republic of Congo (Brazzaville), in Nouabalé-Ndoki National Park (NNNP: 400,000 ha) and the contiguous Kabo forestry concession (267,000 ha). The Republic of Congo is known for its relatively intact forest system, rich in flora and fauna. The region is characterized as tropical lowland forest with highly weathered sandstone, quartzite, and schist bedrock, overlain in places by ancient basin alluvial deposits that have formed well-developed soils [28]. The relief is generally flat, with altitude varying between approximately 350 and 400 meters a.s.l. The climate is dominated by a pronounced dry season, typically beginning at the end of November and extending through early March. Mean annual rainfall is 1700 mm. Minimum and maximum average annual temperatures range between 21.1°–21.9°C and 26.5°–26.8°C. Seven distinct vegetation types characterize the region, with mixed species *terra firma* forest occupying approximately 70% of the area [29]. The forests of NNNP have never been commercially logged, although hunter-gatherer populations are believed to have inhabited the region for approximately 40,000 years, and iron smelting sites, which can seriously degrade forest habitats, are found in the region and have been dated as early as 800 BC [30], [31]. The Kabo concession was selectively logged (<2 trees/ha; [32]) approximately 30 years ago, and is exploited for non-timber forest products by approximately 3,000 people. Combined, the NNNP and the Kabo concessions provide a contiguous, yet heterogeneous landscape in which to evaluate how differences in biotic and abiotic conditions influence patterns of seed and seedling recruitment.

### Site Delineation and Characterization of Seed Rain

We used satellite images to identify forest areas that contained dense mixed, *terra firma* forest in and around Nouabalé-Ndoki National Park. From these potential study areas, we used the geographic survey design component of the Distance 5.0 software [33] to randomly select the location of 30 permanent vegetation plots, distributed over an area of >300,000 ha. The 1-ha (100×100 m) plots were separated by at least 2.5 km to promote independence of samples. From January to May 2005, we tagged, mapped and identified all trees ≥10 cm diameter-at-breast-height (dbh) in each plot. For each tree, we collected three voucher specimens for species verification, measured dbh, and estimated height.

To determine biologically relevant seed densities for seed addition experiments and the pool of species from which to draw our focal species, we quantified the rate and species identity of seed rain within each vegetation plot for one year (May 2005–April 2006) prior to beginning the seed addition experiments, then for a second year (May 2006–June 2007) following seed addition. We quantified seed rain with seed traps (21 per plot, N = 630), which consisted of 1×1 m wooden frames with a mesh center elevated approximately 0.75 m from the ground to avoid seed predation. All fruits and seeds falling into traps were collected, counted, and identified to species at two-week intervals. In total we collected and identified (at least to unique but unknown species) 51,541 mature fruits and 431,770 mature seeds from 428 species.

To facilitate our ability to generalize experimental results from a limited number of species to the broader forest community, we randomly selected five tree species for use in seed addition experiments. Species were chosen from a list of all naturally occurring tree species, with the constraint that we only included species for which at least five seeds occurred in traps during the first year of monitoring (N = 277 species). Constraining the list in this way allowed us to collect sufficient numbers of seeds to conduct the experiment, while not biasing selection towards any particular species characteristic. Focal species varied substantially in terms of regeneration niche, dispersal mode, seed size, and relative abundance (Table 1).

**Table 1:** Characteristics of the focal tree species.

Species and Family	Date of seed addition	Regeneration guild	Fruiting season	Mean conspecific density ha^−1^ (>10 cm dbh)	Median and (maximum) stemdbh (cm)	Dispersalmode	Mean seedlength (cm)	Mean seed rain and (range) (seeds m^−2^)
*Entandophragma utile*Meliaceae	Dec. 2006	NPLD	Sep.–Jan.	1.17	22.0(178.8)	W	0.8	0.10(0–17)
*Pancovia laurentii*Sapindaceae	Jun. 2007	SB	Sep.–Dec.Apr.–May	2.37	24.2(66.9)	P	1.1	0.20(0–39)
*Staudtii kamerunensis*Myristicaceae	Jun. 2007	NPLD	Apr.–May	0.57	43.3(85.1)	P, B	1.9	0.26(0–16)
*Myrianthus arboreus*Urticaceae	Dec. 2006	SB	Apr.–MayOct.–Dec.	3.96	27.1(60.6)	P, B, E	2.1	0.53(0–114)
*Manilkara mabokeensis*Sapotaceae	Dec. 2006	SB	Jul.–Nov.	1.67	19.3118.2	P	1.4	0.58(0–94)

Regeneration guilds include non-pioneer, light demanding (NPLD) and shade bearer (SB). Dispersal mode categories include primate (P; arboreal primates, chimpanzees and gorillas), bird (B), elephant (E), and wind (W). Seed sizes are averaged seed lengths measured from 100 seeds of each species. Average conspecific density was estimated from 30, 1-ha vegetation plots in which stems >10 cm dbh of all tree species were measured, mapped, and identified to species. Seed rain densities are the average densities after two years of monitoring (n = 630 traps).

### Seed Sowing Experiments

We established 63 seed addition “stations” in random locations in 21 of our 30 plots (3 stations per plot). Each station was situated North-South in the plot and consisted of six lines of ten 0.5×0.5 m quadrats, with a 0.5 m buffer maintained around each quadrat. Each quadrat was demarcated with wooden pegs that were replaced as they deteriorated. Here we report on seed addition into 35 quadrats per station; some of the remaining quadrats were caged (see [34]) or left empty because we had originally planned to sow seeds of six species. During installation of the first station, we randomized the assignment of species and seed densities and then used this arrangement in all the other stations to facilitate the identification of quadrats for future monitoring. We then sowed seeds of the five focal species at seven different densities, one density of a single species per quadrat. In this study, we assume that the spatial arrangement of seed augmentation treatments and the order in which species were added to quadrats did not influence the local activity of natural enemies that may have been attracted to these stations.

Densities were multiples (0, 25, 50, 100, 200, 500, and 2000) of the mean natural seed rain density of each species observed in the seed traps the previous year. We added one seed per quadrat when the required augmentation treatment based on natural seed rain densities was less than one. The highest augmentation level exceeded the greatest annual seed rain density in any single seed trap over two years of trap monitoring but is a reasonable representation of seed densities directly under large fruiting trees (Table 1). Seed densities under fruiting canopies were often greater than 1,000 times the average forest-wide densities. Likewise, seed densities in elephant and gorilla dung can greatly surpass forest-wide averages (reaching levels greater than 500 times forest-wide average densities) and even seed dispersal by large birds and arboreal monkeys results in contagious distributions and high densities of conspecific seeds [35]. Sowing seeds at a density 2,000 times the forest-wide average for each species assured that our experiments spanned the entire natural range of seed densities and approached the densities required for site saturation, while remaining biologically plausible. Augmenting seeds over a wide range of densities also facilitated description of the recruitment function [12], permitting quantification of the relative importance of seed versus establishment limitation over essentially any level of seed rain, and helped identify biologically important levels of seed supply compared to other factors (density-dependent and density-independent mortality) in natural communities. Note that to achieve such high densities of seed augmentation, we used relatively small quadrats.

Seed collection and sowing were conducted in the second year of the study (October-December 2005) at the height of the fruiting season for each species. We employed families of indigenous Mbenzélé to widely search the forest for seeds (total search area ∼ 500,000 hectares). The Mbenzélé are hunter-gatherer, semi-nomadic forest people who have an intimate knowledge of the forest, including locations of rare tree species. Approximately 40,000 seeds were collected, cleaned of pulp, screened for insect damage or pathogen infection, and sown into seed addition plots. Damaged and infected seeds were not used. Following seed addition, seedling emergence and mortality were monitored every three months for the first two years of growth. We individually numbered each seedling, attaching a metal tag to the base of the seedlings, and recorded height and number of leaves at each observation period.

### Calculation of Realized Limitation

The failure of a species to recruit at less than maximum density at any given location can be either the result of failure of seeds to arrive or of the lack of suitable conditions for establishment upon arrival. In a heterogeneous natural environment, one would expect a continuum of seed arrival rates and functional traits to jointly influence the relative strengths of seed and establishment limitation. Our seed addition experiment quantifies the number of sites colonized by seedlings when the effect of seed limitation is decreased or eliminated while all other limitations remain in the system. In effect, we quantified *realized* seed and establishment limitation for seedling emergence and survival to the second year of growth [5], [36]. We decouple the strength of realized seed and establishment limitation at each of our seed augmentation levels using a per-seed recruitment effect size as a measure of the relative strength of each process [11].

We calculated an effect size, 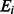, equal to the difference between seedling densities in treatment and control quadrats (0 augmentation level), for the i^th^ augmentation level (i = 1…7), standardized by the number of seeds added to treatment (seed addition) quadrats:
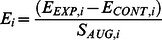
(1)where 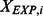 is the number of seedlings in experimental quadrats, 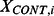 is the number of seedlings in control quadrats, and 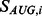 is the number of seeds added to treatment quadrats. This quantity is a measure of the number of new recruits per seed added, after normalizing for background rates of seed arrival and seedling establishment. With this effect size metric, realized seed and establishment limitation are inversely related, occupying opposite ends of a continuum [11], [36]. Realized seed limitation is calculated by the effect size, 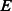. Realized establishment limitation is given by 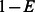. By determining the position of a plant population along this continuum and across multiple levels of seed availability, we can compare the relative strengths of seed and establishment limitation and how they are influenced by seed density, adult conspecific density, site and/or species.

We calculated mean effect sizes of quadrats within each plot to estimate a single plot-level effect size for each species and seed augmentation combination. This effect size is expected to vary between 0 and 1, on average, if density-dependence is weak. By chance, control plots could contain more seedlings than seed addition plots at the end of the experiment, in which case 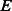 would be negative. 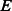 could also be negative if density-dependent mortality is sufficiently strong to result in overcompensation. Finally, if density-dependence is positive and strong, 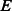 could exceed unity. None of these situations occurred over the course of this study, however; 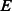 always varied between 0 and 1.

Because seeds for all species were added at equivalent levels relative to species-specific patterns of seed rain, and were added to randomly selected plots, all species and plots should have had equal recruitment probabilities if stochastic processes are dominant. Conversely, significant species-by-plot interactions would provide evidence in support of niche-based models. To examine variation in the strength of realized seed and establishment limitation, we fitted and evaluated generalized linear mixed models (GLMMs) for *E*, with a binomial error distribution and a logit-link. In the GLMM, we treated seed augmentation level and conspecific density as fixed effects, and species, quadrat, and plot as random effects. We also added a crossed random interaction effect of species and plot. Note that we treated species as a random effect because the focal species were chosen randomly from all available species (with the caveats stated above in Site Delineation and Characterization of Seed Rain) and thus represent a random sample from the population of tree species. We were not interested in the particular species, but rather the mean effect of seed and establishment limitation across the tropical community.

These analyses allowed us to determine the relative importance of seed and establishment limitation for the recruitment probability of each individual seed. However, at the population and community level, it may be important to understand whether seed arrival or environmental conditions are better predictors of the resulting seedling density, rather than the individual recruitment probabilities. To evaluate this, we also fit GLMMs to the absolute number of seedlings per seed addition plot, using a lognormal Poisson error distribution to account for over-dispersion and a log-link, with species and plot as crossed random effects. Because sparse data (e.g., low numbers of recruits per plot) and multiple random effects can complicate parameter estimation of this type of likelihood-based technique [37] we used Bayesian inference with Markov Chain Monte Carlo (MCMC) simulation to estimate posterior distributions of model parameters. For both models, we used normally distributed priors for fixed and random effects, and uniform priors on the precisions of the variance components. We fit our models using WinBUGS v. 1.4.1 [38]. For each model, we achieved convergence after 50,000 iterations (the “burn-in”) and based summary statistics on an additional 25,000 iterations. We ran three chains to monitor convergence based on variance components of multiple sequences, and we assessed convergence by visual inspection and with Gelman-Rubin statistics from the R contributed package, coda [39]. For point estimates, we extracted the means of the posterior distributions and derived 95% credible intervals based on the observed percentiles from the MCMC replicates.

### Calculation of Fundamental Limitation

An alternative approach to examine the relative importance of seed and establishment limitation to seedling recruitment is to estimate the degree to which each process would be limiting in the absence of the other - a measure of *fundamental* seed and establishment limitation *sensu* [5]. We define fundamental seed limitation as the difference between the number of seedlings that would recruit to a site if the seed supply were limitless, and post-dispersal constraints to recruitment (e.g., density dependent and independent mortality) are absent [12], relative to the number of seedlings that recruit under ambient conditions. An advantage of estimating seed and establishment limitation in this way is that it allows one to explain why seedlings fail to achieve their “fundamental” optimum (*i.e.*, their potential maximum population size) and it allows one to predict how species and communities might respond to local environmental change [40].

Using the “fundamental limitation” framework to examine the relative importance of seed and establishment limitation to seedling recruitment, and decomposing establishment limitation into density dependent and independent components, assumes that seedling survival is density-dependent and recruitment has an upper limit (*i.e.*, the relationship between seed addition and seedling recruitment must be nonlinear). To estimate fundamental limitation, we first examined whether the assumption of non-linearity between seed augmentation and seedling recruitment was reasonable. To do so, we fit two simple models of seedling recruitment based on the asymptotic Beverton-Holt function. The first model, the *density-dependent model*, is an asymptotic model,
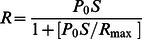
(2)where 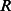 is the number of recruits (seedlings) that emerge from seed input, 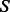. In this study, seed input was the sum of seeds that we sowed and the natural seed rain, 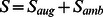, where 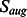 was a multiple of 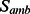. 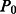 is the proportional recruitment under density-independent mortality only (between 0 and 1) and 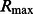
is the maximum number of seedlings if the system were to be saturated with seeds (*i.e.*, density dependence only). The second model, *the density independent model*, is a linear model that is a special case of the first, 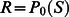 derived by letting 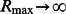. Thus, the first model includes seed limitation, density independence and density dependence, whereas the second model excludes density dependence. By comparing these two models we test whether density significantly influences seedling recruitment. We also tested the two other possible nested models (seed limitation only, 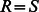, and no density-independent limitation, 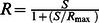, but because both of these models fit the data poorly we exclude them from further discussion.

We fitted the two recruitment models to the seedling data for each species separately and all species combined, for each of the six time periods from three months to two years after seed addition. Models were fitted with PROC NLMIXED in the SAS package, Release 8.2 [41]. We used the default quasi-Newton algorithm to estimate model parameters and 95% confidence intervals, assuming a negative binomial error distribution to our seedling count data (we also tried fitting models with Poisson error, but doing so always resulted in poorer fits). Using the negative binomial error distribution introduces an additional parameter 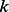, which is an estimate of overdispersion: when 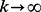, there is no overdispersion and the error distribution collapses into a Poisson; as 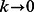, there is added variance in the data (introduced by unmeasured factors) not accounted for by the simpler Poisson. We included vegetation plot as a random effect to assess the degree to which location in the forest affected seedling recruitment relative to the other model parameters. In 11 of 12 model comparisons, the density-dependent model fit the data better than the density-independent model, as assessed by the Akaike Information Criterion, with a differences of four clearly distinguishing the models ([42], Table S1).

We then estimated fundamental limitation using the parameter estimates obtained from the density-dependent, Beverton-Holt model (Equation 2) [12]. The amount of limitation imposed by a single process is calculated by subtracting the number of seedlings that occurred under ambient conditions from the number that would have occurred if the limiting process were removed. Therefore, seed limitation, 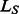, is the difference between the number of seedlings that would emerge if seed supply were limitless and the number of recruits under ambient conditions (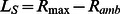). Limitation from density-independence and density-dependence can be found by setting 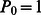 and 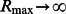, respectively, and comparing these results to seedling recruitment under 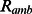. Thus, limitations from density-independent (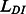) or density-dependent losses (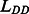) are calculated as: 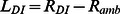 and 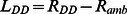. Establishment limitation is represented by the removal of both density-independent and density-dependent losses and can be found by setting 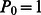 and 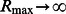 so that 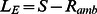. Removal of establishment limitation ensures that all dispersed seeds survive to recruit, *i.e*., that seed input alone determines local abundance.

## Results

**Figure 1:**
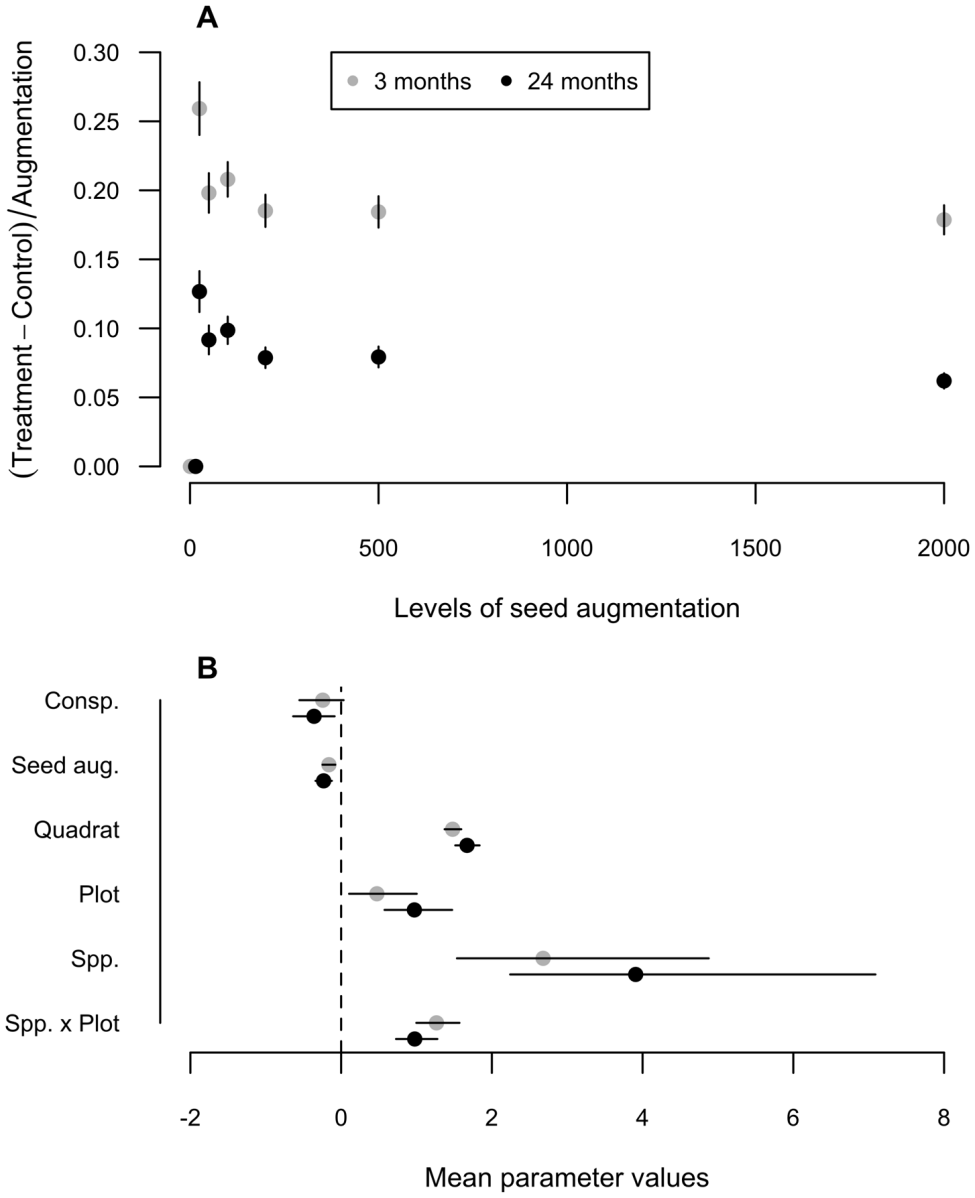
Per-seed recruitment varies across seed augmentation levels and with environmental variation. (A) Per-seed recruitment effect size 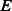 (realized seed and establishment limitation) varies between 0 and 1, with 1 representing complete seed limitation and 0 representing complete establishment limitation. These relatively low effect sizes (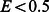) indicate that this natural forest system is more strongly establishment-limited than seed-limited. Error bars are 95% confidence intervals. (B) Results from generalized linear mixed models (GLMM) of per seed recruitment, 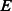, as a function of the seed addition level (Seed aug.) and the density of conspecific trees (Conspecifics) at three and 24 months after seed augmentation. Error bars are the 2.5% and 97.5% credible intervals. Random effects include individual quadrats (Quadrat), the vegetation plot (Plot), the species identification (Species), and species-by-plot interaction (Species × Plot). The significant species-by-plot interactions at both time intervals strongly refute the equivalence of species across a heterogeneous landscape, thereby supporting a niche-based perspective of recruitment.

At local scales, deterministic processes associated with post-dispersal seedling establishment more strongly influenced seedling recruitment than did seed availability. Of sowed seeds, 6389 (16.1%) seedlings recruited after 3 months and 2303 (6.1%) survived to 24 months (Fig. S1 for species-specific results). Per-seed recruitment effect sizes were initially low (*E* = 0.21±0.28 at 3 months; n = 5 species) and decreased over two years (*E* = 0.09±0.20 at 24 months), with a significant reduction in per-seed recruitment over time (linear mixed model: time = −0.006, *t* = −5.96, df = 24, *p*<0.001). Higher initial seed densities resulted in lower per-seed recruitment probabilities, indicating that density dependence at least partially regulates seedling recruitment (Fig. 1a). However, with no significant relationship between seedling recruitment and the density of conspecific adults (Fig. 1b), observed density-dependent mortality was likely a function of direct and/or indirect influences of seeds and seedlings on one another. The position of a species along the establishment - seed limitation continuum was most strongly explained by species identity and the site-by-species interaction term (Fig. 1b; or by site for species-specific models, Fig. S2), suggesting that differences in recruitment probabilities among species vary with site characteristics.

**Figure 2:**
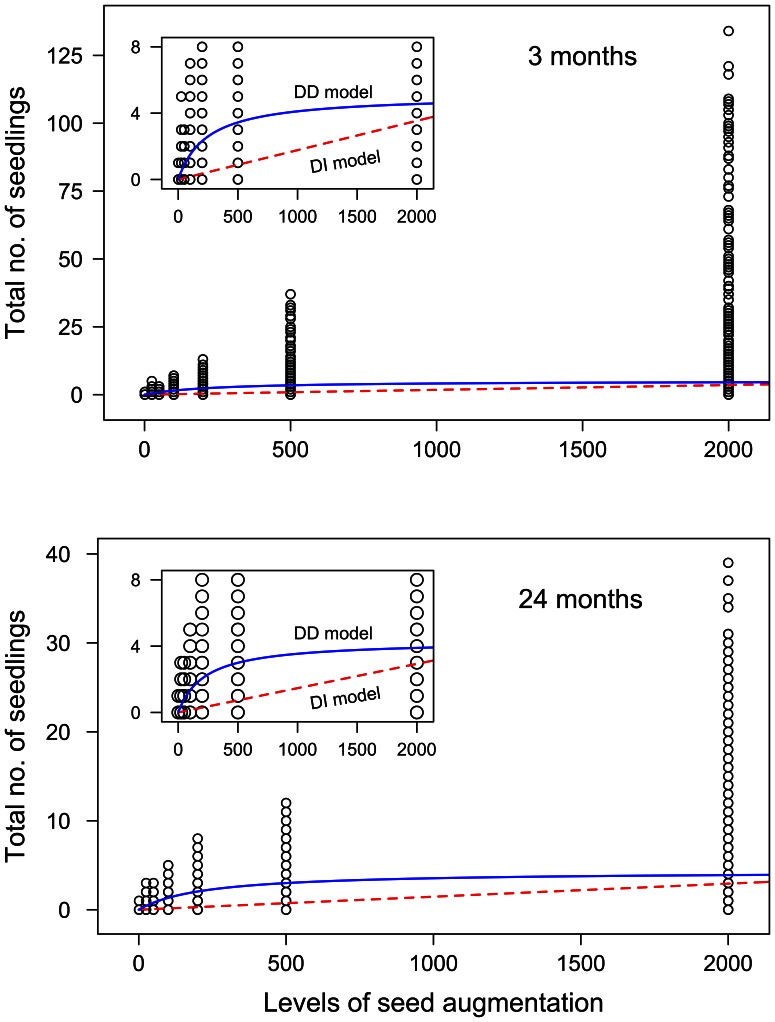
Fit of the two recruitment functions to seed augmentation data. Data are the number of seedlings in each quadrat of all five species at (A) three and (B) 24 months after sowing. The inset graphs emphasize the differences between recruitment functions by magnifying the image (note the truncated y-axis). The dashed line represents the density-independent (DI) model (fitting *P*_0_ and *S*_amb_). The solid line represents the density-dependent (DD) model, accounting for seed-limitation, density-independent limitation, and density-dependent limitation (fitting *P*_0_, *S*_amb_, and *R*_max_). The level of seed augmentation is a multiple of ambient densities observed in nature for each species during the first year of this project. For all species, the density-dependent model provided a better fit than the linear model (see Table S1), providing evidence of density-dependence.

To further elucidate the relative importance of multiple mechanisms in structuring tropical seedling communities, we estimated seed and establishment limitation in the context of fundamental limitation. The *density-dependent* model of seedling recruitment, incorporating both density-independent and density-dependent mortality, fit significantly better than the *density-independent* model for both pooled and species-specific comparisons (Fig. 2, Fig. S3, see Table S1 for comparisons at three months and two years after seed addition). Estimates of proportional recruitment, *P_0_*, and maximum density of recruits, *R_max_*, were similar among pooled and species-specific models. Proportional recruitment ranged from 0.001 to 0.021, and did not significantly change in strength over time (linear mixed model: time = 0.002, *t* = 0.212, df = 24, *p* = 0.83). The maximum density of recruits ranged from 2.9 seedlings m^−2^ (*Manilkara mabokeensis*) to 6.0 seedlings m^−2^ (*Myrianthus arboreus*), with maximum seedling density decreasing significantly over time (linear mixed model: time = −0.087, *t* = −2.33, df = 24, *p* = 0.02). The random effect of plot also increased significantly over time (time = 1.51, *t* = 4.19, df = 24, *p* = 0.0003), demonstrating that the environmental conditions associated with location in the forest were magnified over time in terms of their effects on survival.

Using parameter estimates of the *density-dependent* model (Table S1), we quantified the relative strengths of seed and establishment limitation both for all species pooled together and for individual species. For brevity, we focus on the pooled species results because species-specific estimates of seed and establishment limitation were very similar (Fig. S4).

**Figure 3:**
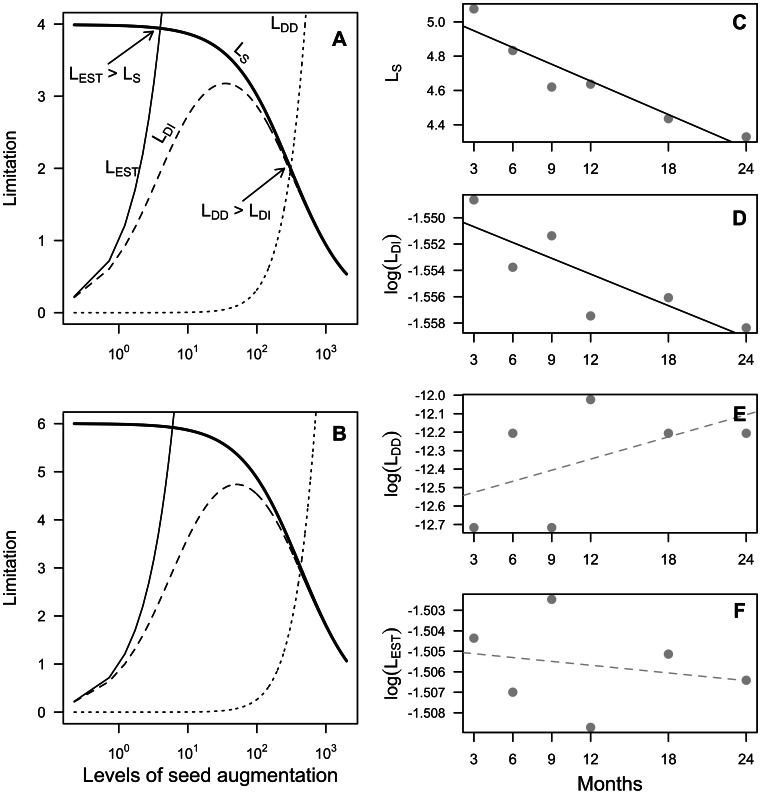
Results of limitation analysis for all species combined. Results are presented for (A) three months and (B) 24 months after seed augmentation. Lines are establishment limitation (L_E_), seed limitation (L_S_), density-dependent mortality (L_DD_), and density-independent mortality (L_DI_). L_E_>L_S_ represents the crossover point at which establishment limitation more strongly limits recruitment than seed limitation. L_DD_>L_DI_ is the point at which density-dependence more strongly limits recruitment than density-independent mechanisms of mortality. The importance of fundamental seed limitation exceeds that of fundamental establishment limitation only at very low seed densities, with crossover points occurring at 4.2 (3 months) and 6.2 (24 months) times ambient seed conditions. These values are well within the range of natural seed rain densities observed across this study site (Table 1). Density-independent mechanisms of seedling mortality more strongly contribute to establishment limitation than do density-dependent mechanisms of mortality until seed densities reach approximately 309 (3 months) and 432 (2 years) times the average ambient seed densities. These values roughly mimic seed densities under parent trees but exceed the observed seed rain densities for most species, thus density-independent factors limit seedling recruitment at most “natural” seed densities whereas density-dependent mechanisms likely control seedling population size at the very high seed densities observed under fruiting canopies. Panels (C–F) depict the correlation of each of the types of limitation over time: black full lines represent a statistically significant correlation; whereas grey dashed lines depict lack of a statistically significant correlation.

Fundamental limitation analysis identified two situations in which seed availability determined the abundance of seedlings, despite the strong niche-based processes identified above. The first occurred under ambient conditions when no seed of a given species was present prior to seed addition. The strength of fundamental seed limitation, L_S_, exceeded that of fundamental establishment limitation, L_E_, only at low seed densities, with crossover points occurring at 5.2 (3 months) and 4.2 (two years) times the average ambient seed densities (Fig. 3). These values are well within the natural range of seed rain densities observed across this study site (∼ 5 times the mean seed rain density, or ∼ 1 seed m^−2^; Table 1). Clearly under this scenario, the importance of seed arrival cannot be ignored – no species can recruit to a site in the complete absence of seed. The removal of this constraint increases seedling recruitment. However, the relative benefit of seed arrival for plant population size declined sharply at approximately 1 seed m^−2^. At this point, a combination of site and species characteristics emerged to more strongly determine the number of recruiting seedlings than the further addition of seeds. Post-dispersal mechanisms of mortality were predominately density-independent, until seed addition treatments reached approximately 305 (3 months) and 426 (2 years) times the average ambient seed densities – seed densities similar to those observed directly under parent trees (Table 1).

**Figure 4:**
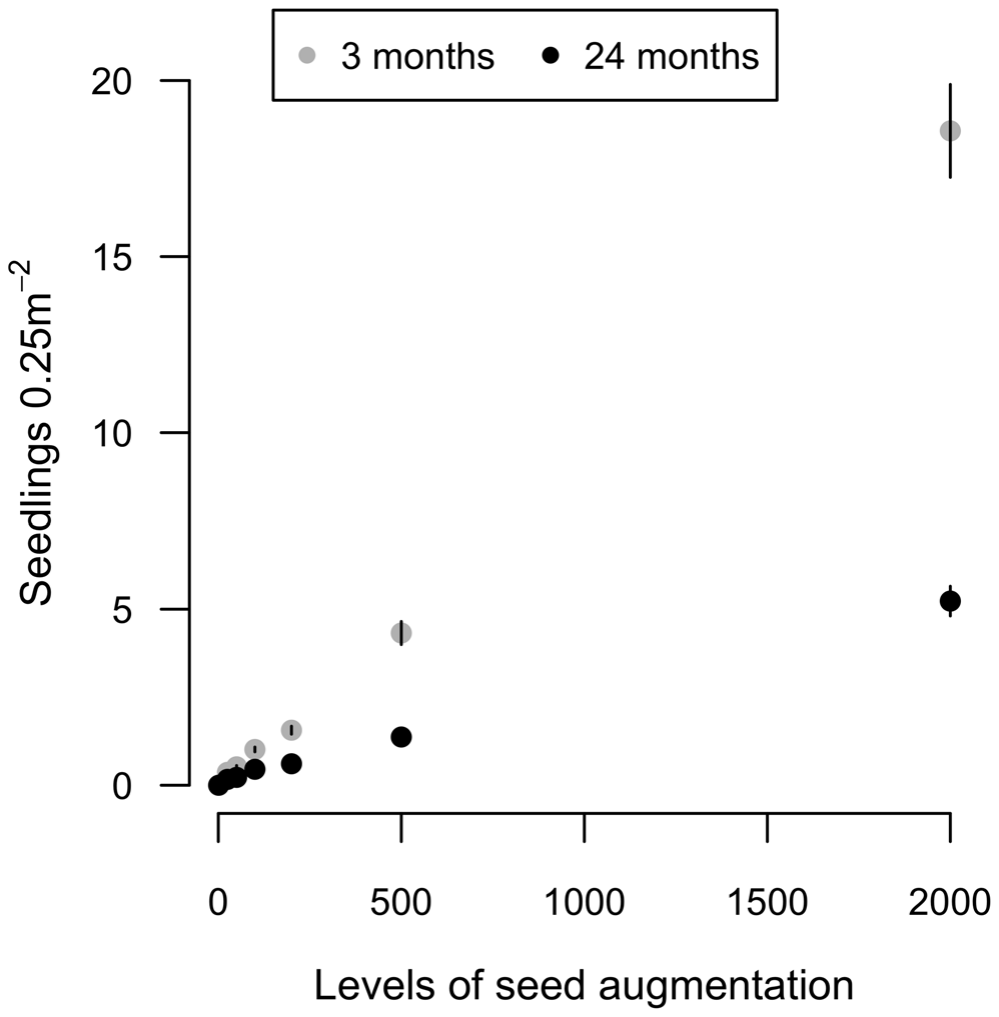
Density of seedlings as a function of seed augmentation level. The densities of seedlings are averaged over 5 species (*Pancovia laurentii, Staudtia kamerunensis, Manilkara mabokeensis, Myrianthus arboreus, and Entandophragma utile)*, as a function of levels of seed augmentation, for the first three months and 24 months of seedling growth. Error bars are 95% confidence intervals. Weak seed limitation observed in Fig. 1a results in a gradual, but significant increase in total seedling numbers at very high seed densities.

Thus, density-independent factors limited seedling recruitment at most seed densities (*i.e.*, those not under parent tree canopies), whereas density-dependent mechanisms likely determined recruitment under parent trees or at sites in which seeds were contagiously deposited. Even though per-seed recruitment probabilities decreased with seed addition and despite evidence of strong density-dependent mortality (Fig. 1a), high initial seed densities overwhelmed density-dependent differences in per seed recruitment so that recruit density was greatest where seeds were most numerous (Fig. 4).

The importance of each process varied with cohort age (Fig. 3). The strengths of seed limitation (*r* = −0.971, *t* = −8.22, df = 4, *p* = 0.001) and density-independent mortality (*r* = −0.877, *t* = −3.65, df = 4, *p* = 0.02) significantly decreased through ontogeny; whereas the strengths of density-dependent mortality (r = 0.569, t = 1.38, df = 4, p = 0.24) and establishment limitation (r = −0.268, t = −0.557, df = 4, p = 0.61) were relatively constant.

## Discussion

With data from a large-scale seed addition experiment, we quantified the relative importance of seed and establishment limitation for seedling recruitment of tropical trees across a heterogeneous Afrotropical forest. Our analyses suggest three general conclusions for trees at our site. First, rates of seedling recruitment are very low and competitive inequality among species largely determines the extent to which limitations in seed arrival will affect community dynamics. Second, seedling recruitment is a result of both dispersal and post-dispersal processes that vary in their relative strengths over time. Through environmental filtering (survival barriers imposed by the abiotic environmental) and niche differentiation (divergence of co-occurring species along environmental axes), niche-based processes eliminate most gains in recruitment from seed addition [43], [44]. Third, dispersal contributes most to seedling populations in two situations: when there are almost no seeds on the forest floor and when there are very high densities of seeds. Under ambient conditions, seed arrival increases seedling densities, despite strong establishment limitation that kills most seeds and seedlings. At extremely high seed densities, seed arrival can overwhelm niche-based processes that limit establishment, resulting in greater numbers of seedlings up to two years after seed arrival.

### By Dispersal or by Niche: The Relative Strengths of Seed and Establishment Limitation

Both dispersal and niche-based processes influence the early stages of establishment and survival in our seedling community. Dispersal contributed to the initial distributions of individuals and species. As demonstrated in previous seed addition studies [10], [11], [45], [46], the arrival of seeds (addition into experimental stations) increased the density of tree seedlings. Dispersal effects appear to be important, but only within the confines of environmental gradients. Stated differently, it matters where seeds arrive: variation in abiotic factors (e.g., soils, nutrients, sunlight) and biotic factors (e.g., seed and seedling predation) limit rates of germination and early recruitment (e.g., [34]). In our study system these mortality factors reduced early recruitment to 17% of all arrived seeds. Once a seedling is established, post-dispersal mortality factors modify the initial distributions of individuals and species. Similar to studies from the Neotropics [20], the impact of seed addition declined over two years following germination so that only 6.1% of seeds survived to two years. Thus, processes of dispersal, abiotic environmental filtering, and niche differentiation interact to jointly determine community assembly.

The relative importance of these processes depends on the definition of limitation that is applied. Using the framework of *realized limitation*, establishment limitation would be considered stronger than seed limitation, with mortality of 94% of seeds within two years. On a per-seed basis, more seeds are killed than survive; thus, post-arrival mortality should drive population size. Using the framework of *fundamental limitation*, on the other hand, seed limitation would be considered more important as the addition of seeds led to higher population sizes of our focal species. Across our sites, mean background seed density was less than one seed per m^2^ for all five randomly selected species (Table 1). Because ambient seed density is so low for those species, seed arrival has a much greater effect on population size than removing mortality sources, even though few of the new seedlings survive due to density-independent mortality. Simply put, without seed arrival, mortality factors have little to act on; thus the addition of seeds increases the population size more than eliminating sources of mortality. Here we emphasize fundamental limitation because it better represents the dynamics of tropical tree communities, where most species are exceedingly rare – often represented by a single mature tree at local scales (<2 adults ha^−1^ in this study; e.g., [47]).

To better understand where dispersal or niche-based processes dominate, and the conditions that influence their joint roles, we applied models of fundamental limitation to our experimental data to quantify the relative strengths of these forces, to partition establishment limitation into density-independent and density-dependent processes, and to evaluate their temporal dynamics (e.g., [12], [46]). Seed arrival and environmental conditions impose complex and dynamic forces on the seedling community. At ambient conditions, the strength of seed limitation decreased significantly over time as niche-based processes erased its input into the system. Consequently, the strength of density-independent mortality decreased significantly as the number of seedlings to act on decreased. Density-dependent mortality became increasingly more important over time (although not significantly so, in our case), perhaps as seedlings become larger and more likely to interact with neighbors or more visible to predators. Therefore we suggest for our site that processes driving establishment limitation change over time, with environmental filtering being replaced by niche differentiation.

### Windows of Opportunity for Dispersal Processes

Using models of fundamental limitation, we identified two windows in which dispersal plays a stronger role in dictating patterns of seedling recruitment than do niche-based processes. At low seed densities (<1 seed m^−2^), seed limitation influenced recruitment more than establishment limitation. The removal of this constraint increases seedling recruitment. However, the relative benefit of seed arrival for plant population size declined sharply at densities greater than 1 seed m^−2^. Establishment limitation is likely to dominate where seed densities are greater than 1 seed m^−2^, such as the local neighborhood to tree and areas of high conspecific tree density. Seed densities greater than 1 m^−2^ occurred up to at least 20 m for several monkey- and bird-dispersed Central African tree species, and well beyond 20 m for small-seeded, wind-dispersed tree species [48]. In Cameroon, establishment limitation was consistently two to four times stronger than seed limitation for the grove-forming species, *Microberlinia bisulcata*, with adult stem density and seed rain densities (4.96–5.45 seeds m^−2^ from seed traps) much higher than our randomly selected species, [46].

At extremely high seed densities, those found directly under fruiting tree canopies or sites of contagious dispersal [35], we observed a second window through which dispersal processes can overcome niche-based processes. In our study, post-dispersal mechanisms of mortality were predominately density-independent, until seed addition treatments reached seed densities of 30–180 seeds m^−2^, at which point density-dependent mortality dominated. Based on natural variation in seed rain, Wright et al. [49] found density-dependent mortality to constrain recruitment at much lower seed densities. Additional research is necessary to evaluate whether density-dependence operates at higher densities at our site or, alternatively, whether the relatively small size of our quadrats (0.5×0.5 m) diminished its importance (e.g., if the small scale reduced the probability of predators finding seeds). Even though per-seed recruitment probabilities decreased with seed addition and despite evidence of strong density-dependent mortality (Fig. 3a, b), high initial seed densities overwhelmed density-dependent differences in per seed recruitment so that recruit density was greatest where seeds were most numerous (Fig. 4). This may be evidence of satiation of seed predators. Caging experiments at our study site demonstrate that seed and seed predation by vertebrates strongly reduce seedling recruitment [34]. Satiation of predators could be an effective and widespread reproductive strategy for tropical trees, as recent studies demonstrate that masting species are relatively common (23–50% of tree species) in some forests [49], [50]. Alternatively, incomplete searching by generalist predators would also allow seedlings to escape mortality. Either way, it may be that these early gains from high seed input are ephemeral and will be lost over time with niche-based processes ultimately prevailing.

Density-dependent mortality could be due to either direct influences, like competition among seedlings, or indirect influences, such as density-responsive predators. Direct interactions among seedlings are generally assumed to be unlikely in tropical forests because of the low density and small stature of seedlings [51], [52], although in a study of 163 species, Metz *et al.* [53] found conspecific seedling density to negatively impact first year survival. Large aggregations of seeds could have attracted generalist or specialist seed predators or fungi that behave in a density-responsive manner [27], [54]. Given that we found no statistically significant relationship between seedling recruitment and the density of conspecific adults, we attribute density-dependent mortality to generalist predators. In a concurrent study in which we caged seeds and seedlings to protect them from vertebrate predation, we similarly found no significant relationship between the distance or density of conspecific trees and the probability of seedling establishment or survival [34].

### Concluding Remarks

Our study underscores the dual effects of both dispersal and niche-based processes in structuring plant communities [20], [55], [56], [57], [58]. It is unique in that it quantifies the relative importance of these processes for tree seedlings and plant communities under a range of natural conditions and through time. Compared to studies that non-randomly choose focal species or that are limited to narrow habitat types, the random selection of tree species and the extensive and heterogeneous area over which we added seeds allowed us to capture the range of variation in nature. The use of greater than 5 randomly selected species would have improved our ability to generalize results more broadly. Furthermore, we recognize the outcome of early establishment processes needs to be seen in the context of later stages of tree dynamics, a challenge that necessitates long-term monitoring studies. As we continue to monitor these seedling plots, we expect the strength of niche differentiation will continue to reduce the effect of seed arrival over the lifetime of individuals, increasing the relative importance of niche-based processes.

In summary, our results demonstrate that in a Central African forest the probability of recruitment of tropical tree seedlings varies with species identity and environmental characteristics of forest sites. Dispersal does predominate under specific conditions; but the importance of seed dispersal may be short-lived as niche-based mortality factors diminish the number of recruits. Time will tell whether seed addition results in significantly higher numbers of trees in treatment plots than control plots. The importance of niche-based processes in this tropical forest raises the question of what we should expect in ecosystems that have much stronger resource heterogeneity. Understanding diverse community assemblages may best be advanced by assessing the conditions under which these processes shift in strength. Doing so gains practical importance when environmental changes associated with industrial logging, agriculture and climate change necessitate deliberate management for species diversity.

## Supporting Information

Figure S1 **Species-specific estimates of (a) Per seed recruitment effect size, *E*, and (b) seedling densities at three months and 24 months after seeds were sowed at six levels.** These relatively low effect sizes (E <0.5) indicate that this natural forest system is more strongly establishment limited than seed limited. The species include *Pancovia laurentii* (Pala), *Staudtia kamerunensis* (Stka), *Manilkara mabokeensis* (Mama), *Myrianthus arboreus* (Myar), and *Entandophragma utile* (Enut). Error bars are 95% confidence intervals. Weak seed limitation results in a gradual, but significant increase in total seedling numbers at very high seed densities.
(PDF)

Figure S2 **Species specific results from generalized linear mixed models (GLMM) on (a) per seed effect size, *E*, and (b) number of seedlings as a function of seed addition level (Seed aug.) and the density of conspecific trees (Conspecifics) after three months and 24 months after seed augmentation.** Error bars are 95% credible intervals. Random effects include individual quadrats (Individual), the vegetation plot (Plot), the species identification (Species), and species by plot interaction (Species × Plot). The species include *Pancovia laurentii* (Pala), *Staudtia kamerunensis* (Stka), *Manilkara mabokeensis* (Mama), *Myrianthus arboreus* (Myar), and *Entandophragma utile* (Enut).
(PDF)

Figure S3 **Fit of the recruitment functions to species-specific seed augmentation data.** Each of the panels depicts the recruitment function of a species at either (a) three months or (b) 24 months after sowing, with species including: *Pancovia laurentii* (Pala), *Staudtia kamerunensis* (Stka), *Manilkara mabokeensis* (Mama), *Myrianthus arboreus* (Myar), and *Entandophragma utile* (Enut). The dashed line represents the *density-independent* (DI) model (fitting *P*_0_ and *S*_amb_) and the solid line represents the *density-dependent* (DD) model (fitting *P*_0_, *S*_amb_, and *R*_max_). The level of seed augmentation is a multiple of ambient densities observed in nature for each species during the first year of this project. For all species, the full Beverton-Holt model (DD) provided an improved fit to the linear model (Table S1).
(PDF)

Figure S4
**Results of limitation analysis for five tropical tree species, *Entandophragma utile* (Enut), *Manilkara mabokeensis* (Mama), *Myrianthus arboreus* (Myar), *Pancovia laurentii* (Pala), and *Staudtia kamerunensis* (Stka), at (a) three months and (b) 24 months after seed augmentation.** Lines are establishment limitation (blue), seed limitation (black), density-dependent mortality (black), and density-independent mortality (green). For all species, establishment limitation becomes a stronger source of recruitment limitation than seed limitation (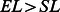) at very low seed input levels (4–6 times mean ambient seed densities). The strength of seed limitation declines sharply at seed addition levels below 1 seed m^−2^ (0.16–0.98 seeds m^−2^). For all species, density-independent mechanisms of seedling mortality more strongly prevent seedlings from achieving maximum population densities than density-dependent mechanisms until seed availability reaches high addition levels (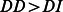, 222–765 times mean ambient seed rain densities). For four of five species, the point at which density-dependence more strongly limits seedling recruitment than either seed limitation or density-independent factors occurs at seed densities within the range observed in seed trap.(PDF)

Table S1
**Parameter values from the density-independent (DI) model and density-dependent (DD) models for pooled and individual species.** The DI model includes parameters for density-independent mortality, *P*_0_, overdispersion, 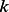, and the random effect of *Plot*. The DD model includes a parameter for the maximum number of seedlings, *R*_max_. The CIs for each parameter are 95% credible intervals. When parameter results of DD model between three months (3 mo.) and 24 months (24 mo.) are compared, the DI parameter usually differs from 0 at three months, but not after 24 months, suggesting that the effect of seed addition begins to disappear by 2 years. Species codes are *Pancovia laurentii* (Pala), *Staudtia kamerunensis* (Stka), *Manilkara mabokeensis* (Mama), *Myrianthus arboreus* (Myar), *and Entandophragma utile* (Enut). Bolded AIC values highlight the best model for each Species and time combination, based on a difference of at least four.(DOCX)

## References

[1] Chesson PL (1985) Coexistence of competitors in spatially and temporally varying environments – a look at the combined effects of different sorts of variability. Theor Popul Biol 28: 263–287.

[2] Hurtt GC, Pacala SW (1995) The consequences of recruitment limitation- reconciling chance, history and competitive differences between plants. J Theor Biol 176: 1–12.

[3] Tilman D (1994) Competition and biodiversity in spatially structured habitats. Ecology 75: 2–16.

[4] Clark JS, Beckage B, Camill P, Cleveland B, HilleRisLambers J, et al (1999) Interpreting recruitment limitation in forests. Am J Bot 86: 1–16. 21680341

[5] Nathan R, Muller-Landau HC (2000) Spatial patterns of seed dispersal, their determinants and consequences for recruitment. Trends Ecol Evol 15: 278–285. 10856948 10.1016/s0169-5347(00)01874-7

[6] Chambers JC, Macmahon JA (1994) A day in the life of a seed - movements and fates of seeds and their implications for natural and managed systems. Annu Rev Ecol Syst 25: 263–292.

[7] Swaine MD (1996) The ecology of tropical forest tree seedlings. Parthenon, New York, New York, USA.

[8] Turnbull LA, Crawley MJ, Rees M (2000) Are plant populations seed-limited? A review of seed sowing experiments. Oikos 88: 225–238.

[9] Eriksson O, Ehrlen J (1992) Seed and microsite limitation of recruitment in plant populations. Oecologia 91: 360–364. 28313543 10.1007/BF00317624

[10] Svenning JC, Wright SJ (2005) Seed limitation in a Panamanian forest. J Ecology 93: 853–862.

[11] Clark CJ, Poulsen JR, Levey DJ, Osenberg CW (2007) Are plant populations seed limited? A critique and meta-analysis of seed addition experiments. Am Nat 170: 128–142. 17853997 10.1086/518565

[12] Poulsen JR, Osenberg CW, Clark CJ, Levey DJ, Bolker BM (2007) Plants as reef fish: Fitting the functional form of seedling recruitment. American Naturalist 170: 167–183. 10.1086/518945 17874368

[13] Chave J (2004) Neutral theory and community ecology. Ecology Letters 7: 241–253.

[14] Cornell HV, Lawton JH (1992) Species interactions, local and regional processes, and limits to the richness of ecological communities: a theoretical perspective. J Anim Ecol 61: 1–12.

[15] Hurtt GC, Pacala SW (1995) The consequences of recruitment limitation- reconciling chance, history and competitive differences between plants. J Theor Biol 176: 1–12.

[16] Hubbell SP (2001) The Unified Neutral Theory of biodiversity and Biogeography. Princeton University Press, Princeton, New Jersey.

[17] Grubb PJ (1977) Maintenance of species richness in plant communities - importance of regeneration niche. Biol Rev 52: 107–145.

[18] Kraft NJB, Valencia R, Ackerly DD (2008) Functional Traits and Niche-Based Tree Community Assembly in an Amazonian Forest. Science 322: 580–582. 18948539 10.1126/science.1160662

[19] Muller-Landau HC, Levin SA (2010) The tolerance-fecundity trade-off and the maintenance of diversity in seed size. Proc Nat Acad Sci 107: 4242–4247. 20160078 10.1073/pnas.0911637107 PMC2840174

[20] Paine CET, Harms KE (2009) Quantifying the effects of seed arrival and environmental conditions on tropical seedling community structure. Oecologia 160: 139–150. 19142667 10.1007/s00442-008-1269-6

[21] Clark JS (2008) Beyond neutral science. Trends Ecol Evol 24: 8–15. 19026462 10.1016/j.tree.2008.09.004

[22] Chesson PL, Warner RR (1981) Environmental variability promotes coexistence in lottery competitive systems. Am Nat 117: 923–943.

[23] Condit R, Hubbell SP, Foster RB (1994) Density dependence in two understory tree species in a neotropical forest. Ecology 75: 671–680.

[24] Connell JH (1971) On the role of natural enemies in preventing competitive exclusion in some marine animals and rain forest trees. In: Boer PJD, Gradwell GR, editors, Dynamics of populations. Center for Agricultural Publication and Documentation, Wageningen, 298–312.

[25] Harms KE, Wright SJ, Calderon O, Hernandez A, Herre EA (2000) Pervasive density-dependent recruitment enhances seedling diversity in a tropical forest. Nature 404: 493–495. 10761916 10.1038/35006630

[26] Hubbell SP, Condit R, Foster RB (1990) Presence and absence of density dependence in a neotropical tree community. Philos T Roy Soc B 330: 269–281.

[27] Janzen DH (1970) Herbivores and number of tree species in tropical forests. Am Nat 104: 501–528.

[28] Lanfranchi R, Schwartz D (1991) Les remaniements des sols pendant le Quaternaire supérieur au Congo. II : Evolution des paysages dans la région du Mayombe. Cahiers ORSTOM, série Pédologie.

[29] Harris DJ (2002) The vascular plants of the Dzanga-Sangha Reserve, Central African Republic. Scripta Botanica Belgica.

[30] Zangato ME (1999) African archaeology. Sociétés Préhistoriques et Mégalithes dans le Nord-Ouest de la République Centrafricaine. BAR, Cambridge, UK.

[31] Lanfranchi R, Ndanga J, Zana H (1998) New carbon 14C datings of iron metallurgy in the Central African dense forest. In: Resource use in the Trinational Sangha river region of Equatorial Africa: histories, knowledge forms, and institutions (ed. Eves, HE) Yale School of Forestry and Environmental Studies, New Haven, CT, 41–50.

[32] Congolaise Industrielle des Bois (2006) Plan d’amenagement de l’unité forestière d’aménagement de Kabo (2005–2034). Ministry of Forest Economy, Brazzaville.

[33] Thomas L, Laake JL, Strindberg S, Marques FFC, Buckland ST, et al.. (2006) Distance 5.0. Research Unit for Wildlife Population Assessment, University of St. Andrews, St. Andrews, UK.

[34] Clark CJ, Poulsen JR, Levey DJ (2012) Vertebrate herbivory impacts seedling recruitment more than niche partitioning, distance- or density-dependent mortality. Ecology 93: 554–564. 22624210 10.1890/11-0894.1

[35] Clark CJ, Poulsen JR, Connor EF, Parker VT (2004) Fruiting trees as dispersal foci in a semi-deciduous tropical forest. Oecologia 139: 66–75. 14745649 10.1007/s00442-003-1483-1

[36] Muller-Landau HC, Wright SJ, Calderon O, Hubbell SP, Foster RB (2002) Assessing recruitment limitation: concepts, methods and case-studies from a tropical forest. In Levey DJ, Galetti M, editors, Seed Dispersal and Frugivory: Ecology, Evolution and Conservation. CAB International, Wallingford, 35–54.

[37] Bolker BM, Brooks ME, Clark CJ, Geange SW, Poulsen JR, et al (2009) Generalized linear mixed models: a practical guide for ecology and evolution. Trends Ecol Evol 24: 127–135. 19185386 10.1016/j.tree.2008.10.008

[38] Spiegelhalter DJ, Thoomas A, Best NG, Lunn D (2003) WinBUGS Version 1.4. Imperial College and MRC Biostatistics Unit: UK.

[39] Plummer M, Best NG, Cowles NK, Vines SK (2005) coda: output analysis and diagnostics for MCMC. R package, version 0.9–5.

[40] McGill BJ, Enquist BJ, Weiher E, Westoby M (2006) Rebuilding community ecology from functional traits. Trends Ecol Evol 21: 178–185. 16701083 10.1016/j.tree.2006.02.002

[41] SAS Institute Inc (2001) SAS/STAT Software: Changes and Enhancements, Release 8.2. Cary, NC.

[42] Bolker BM (2008) Ecological Models and Data in R. Princeton University Press, Princeton, NJ.

[43] Silvertown J (2004) Plant coexistence and the niche. Trends Ecol Evol 19: 605–611.

[44] Cornwell WK, Schwilk DW, Ackerly DD (2006) A trait-based test for habitat filtering: Convex hull volume. Ecology 87: 1465–1471. 16869422 10.1890/0012-9658(2006)87[1465:attfhf]2.0.co;2

[45] Makana J-R, Thomas SC (2004) Dispersal limits natural recruitment of African mahoganies. Oikos 106: 67–72.

[46] Norghauer JM, Newbery DM (2010) Recruitment limitation after mast-seeding in two African rain forest trees. Ecology 91: 2303–2312. 20836452 10.1890/09-0071.1

[47] Hubbell SP (1979) Tree dispersion, abundance, and diversity in a tropical dry forest. Science 203: 1299–1309. 17780463 10.1126/science.203.4387.1299

[48] Clark CJ, Poulsen JR, Bolker BM, Connor EF, Parker VT (2005) Comparative seed shadows of bird-, monkey-, and wind-dispersed trees. Ecology 86: 2684–2694.

[49] Wright SJ, Muller-Landau HC, Calderon O, Hernandez A (2005) Annual and spatial variation in seedfall and seedling recruitment in a Neotropical forest. Ecology 86: 848–860.

[50] Norden N, Chave J, Belbenoit P, Caubère A, Châtelet P, et al (2007) Mast Fruiting Is a Frequent Strategy in Woody Species of Eastern South America. PLoS ONE 210: e1079 doi:10.1371/journal.pone.0001079. 10.1371/journal.pone.0001079 PMC2031917 17957261

[51] Paine CET, Harms KE, Schnitzer SE, Carson WP (2008) Weak competition among tropical tree seedlings: Implications for species coexistence. Biotropica 40: 432–440.

[52] Svenning JC, Fabbro T, Wright SJ (2008) Seedling interactions in a tropical forest in Panama. Oecologia 155: 143–150. 17965886 10.1007/s00442-007-0884-y

[53] Metz MR, Sousa WP, Valencia R (2010) Widespread density-dependent seedling mortality promotes species coexistence in a highly diverse Amazonian rain forest. Ecology 91: 3675–3685. 21302838 10.1890/08-2323.1

[54] Bell T, Freckleton RP, Lewis OT (2006) Plant pathogens drive density-dependent seedling mortality in a tropical tree. Ecol Lett 9: 569–574. 16643302 10.1111/j.1461-0248.2006.00905.x

[55] Adler PB, HilleRisLambers J, Levine JM (2007) A niche for neutrality. Ecol Lett 10: 95–104. 17257097 10.1111/j.1461-0248.2006.00996.x

[56] Chase JM (2003) Community assembly: when should history matter? Oecologia 136: 489–498. 12836009 10.1007/s00442-003-1311-7

[57] Karst J, Gilbert B, Lechowicz MJ (2005) Fern community assembly: The roles of chance and the environment at local and intermediate scales. Ecology 86: 2473–2486.

[58] Shipley B, Paine TEC, Baraloto C (2012) Quantifying the importance of local niche-based and stochastic processes to tropical tree community assembly. Ecology 93: 760–769. 22690627 10.1890/11-0944.1

