# Supplementary material for: Roles of Seed and Establishment Limitation in Determining Patterns of Afrotropical Tree Recruitment
Source: PLoS One. 2013 May 14;8(5):e63330. doi: 10.1371/journal.pone.0063330 (PMC3653939; doi:10.1371/journal.pone.0063330)
Supplement: Figure S1 — Species-specific estimates of (a) Per seed recruitment effect size, E, and (b) seedling densities at three months and 24 months after seeds were sowed at six levels. These relatively low effect sizes (E <0.5) indicate that this natural forest system is more strongly establishment limited than seed limited. The species include Pancovia laurentii (Pala), Staudtia kamerunensis (Stka), Manilkara mabokeensis (Mama), Myrianthus arboreus (Myar), and Entandophragma utile (Enut). Error bars are 95% confidence intervals. Weak seed limitation results in a gradual, but significant increase in total seedling numbers at very high seed densities. (PDF) [file pone.0063330.s001.pdf]

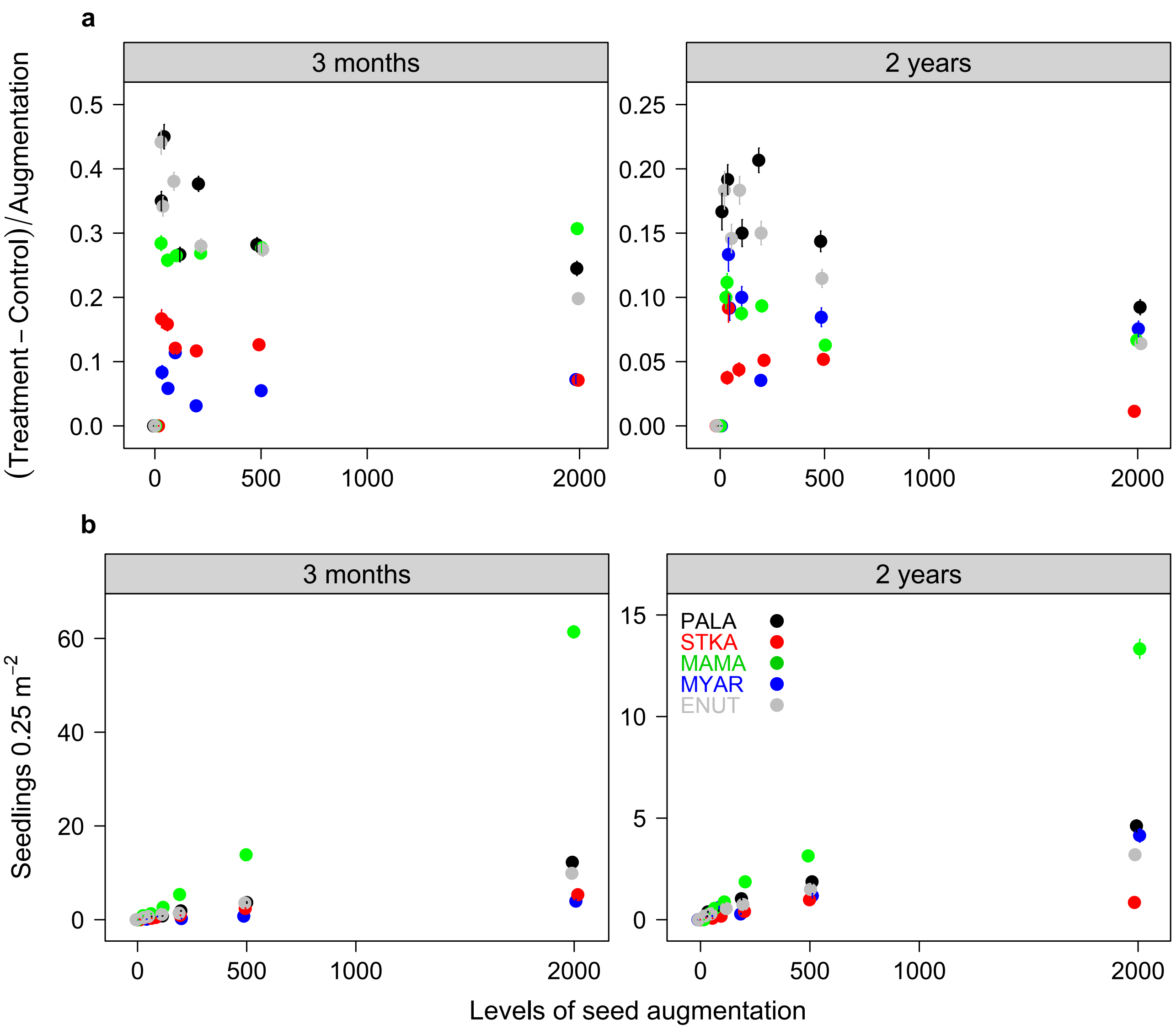

Figure S1. Species-specific estimates of (a) Per seed recruitment effect size, *E*, and (b) seedling densities at three months and 24 months after seeds were sowed at six levels. These relatively low effect sizes (E < 0.5) indicate that this natural forest system is more strongly establishment limited than seed limited. The species include *Pancovia laurentii* (Pala), *Staudtia kamerunensis* (Stka), *Manilkara mabokeensis* (Mama), *Myrianthus arboreus* (Myar), and *Entandophragma utile* (Enut). Error bars are 95% confidence intervals. Weak seed limitation results in a gradual, but significant increase in total seedling numbers at very high seed densities.
