# Supplementary material for: Roles of Seed and Establishment Limitation in Determining Patterns of Afrotropical Tree Recruitment
Source: PLoS One. 2013 May 14;8(5):e63330. doi: 10.1371/journal.pone.0063330 (PMC3653939; doi:10.1371/journal.pone.0063330)
Supplement: Figure S2 — Species specific results from generalized linear mixed models (GLMM) on (a) per seed effect size, E, and (b) number of seedlings as a function of seed addition level (Seed aug.) and the density of conspecific trees (Conspecifics) after three months and 24 months after seed augmentation. Error bars are 95% credible intervals. Random effects include individual quadrats (Individual), the vegetation plot (Plot), the species identification (Species), and species by plot interaction (Species × Plot). The species include Pancovia laurentii (Pala), Staudtia kamerunensis (Stka), Manilkara mabokeensis (Mama), Myrianthus arboreus (Myar), and Entandophragma utile (Enut). (PDF) [file pone.0063330.s002.pdf]

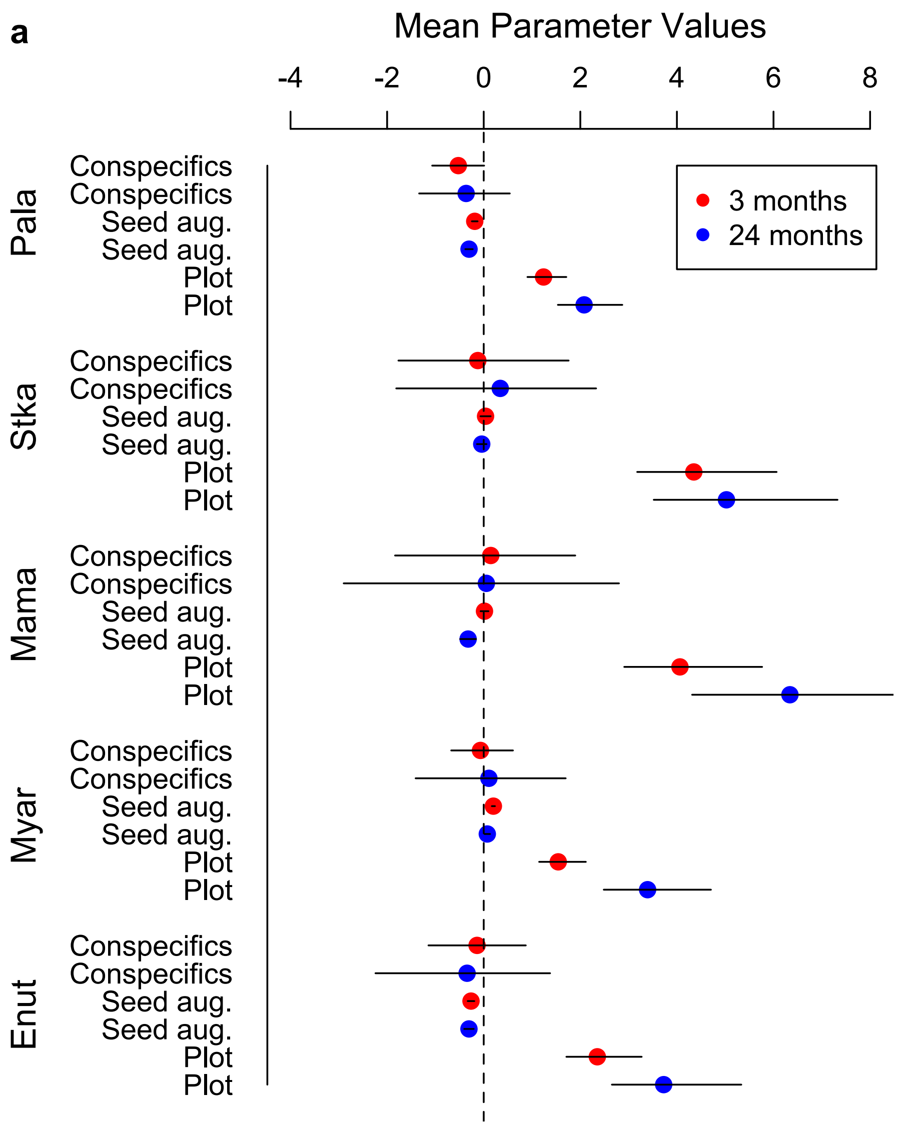
a
Mean Parameter Values
-4
-2
0
2
4
6
8
3 months
24 months
Pala
Stka
Mama
Myar
Enut
Conspecifics
Conspecifics
Seed aug.
Seed aug.
Plot
Plot

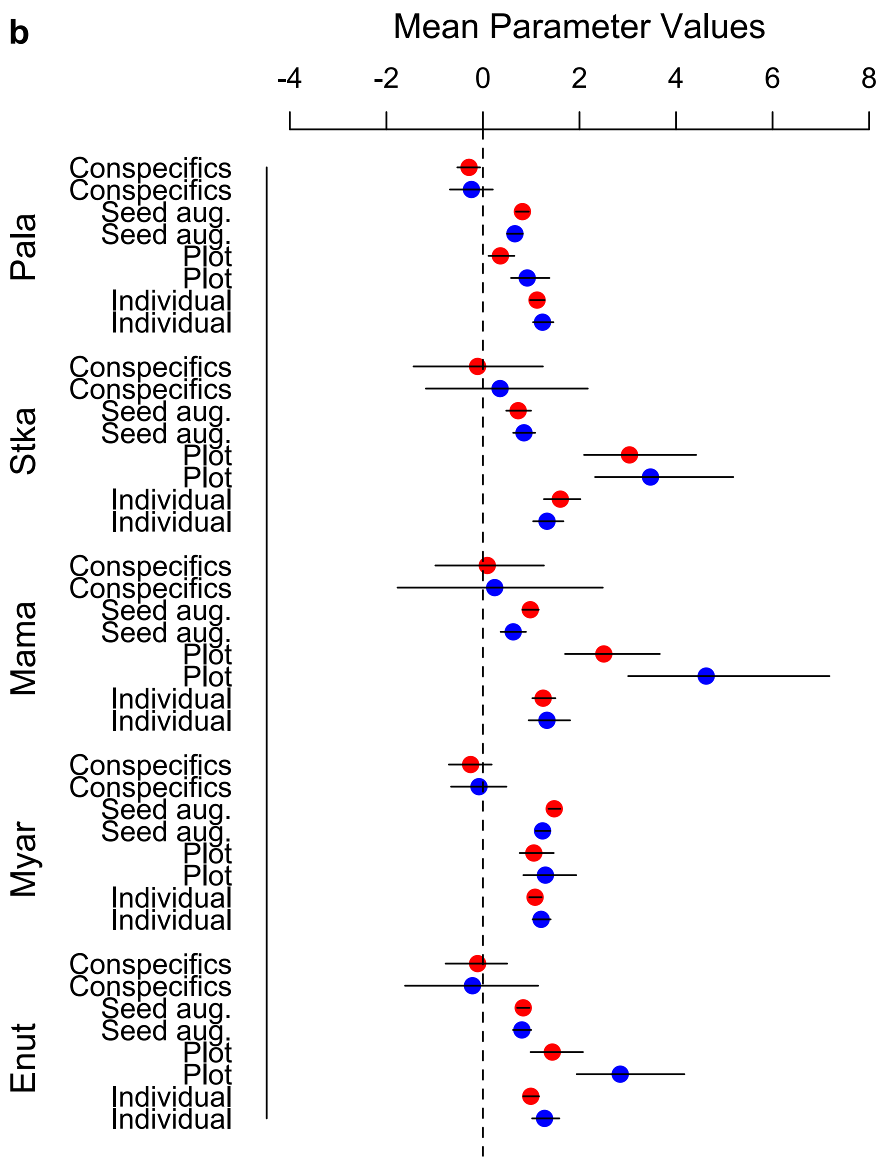
b
Mean Parameter Values
-4
-2
0
2
4
6
8
Pala
Stka
Mama
Myar
Enut
Conspecifics
Conspecifics
Seed aug.
Seed aug.
Plot
Plot
Individual
Individual

Figure S2. Species specific results from generalized linear mixed models (GLMM) on (a) per seed effect size, *E*, and (b) number of seedlings as a function of seed addition level (Seed aug.) and the density of conspecific trees (Conspecifics) after three months and 24 months after seed augmentation. Error bars are 95% credible intervals. Random effects include individual quadrats (Individual), the vegetation plot (Plot), the species identification (Species), and species by plot interaction (Species x Plot). The species include *Pancovia laurentii* (Pala), *Staudtia kamerunensis* (Stka), *Manilkara mabokeensis* (Mama), *Myrianthus arboreus* (Myar), and *Entandophragma utile* (Enut).
