# Supplementary material for: Roles of Seed and Establishment Limitation in Determining Patterns of Afrotropical Tree Recruitment
Source: PLoS One. 2013 May 14;8(5):e63330. doi: 10.1371/journal.pone.0063330 (PMC3653939; doi:10.1371/journal.pone.0063330)
Supplement: Figure S3 — Fit of the recruitment functions to species-specific seed augmentation data. Each of the panels depicts the recruitment function of a species at either (a) three months or (b) 24 months after sowing, with species including: Pancovia laurentii (Pala), Staudtia kamerunensis (Stka), Manilkara mabokeensis (Mama), Myrianthus arboreus (Myar), and Entandophragma utile (Enut). The dashed line represents the density-independent (DI) model (fitting P 0 and S amb) and the solid line represents the density-dependent (DD) model (fitting P 0, S amb, and R max). The level of seed augmentation is a multiple of ambient densities observed in nature for each species during the first year of this project. For all species, the full Beverton-Holt model (DD) provided an improved fit to the linear model (Table S1). (PDF) [file pone.0063330.s003.pdf]

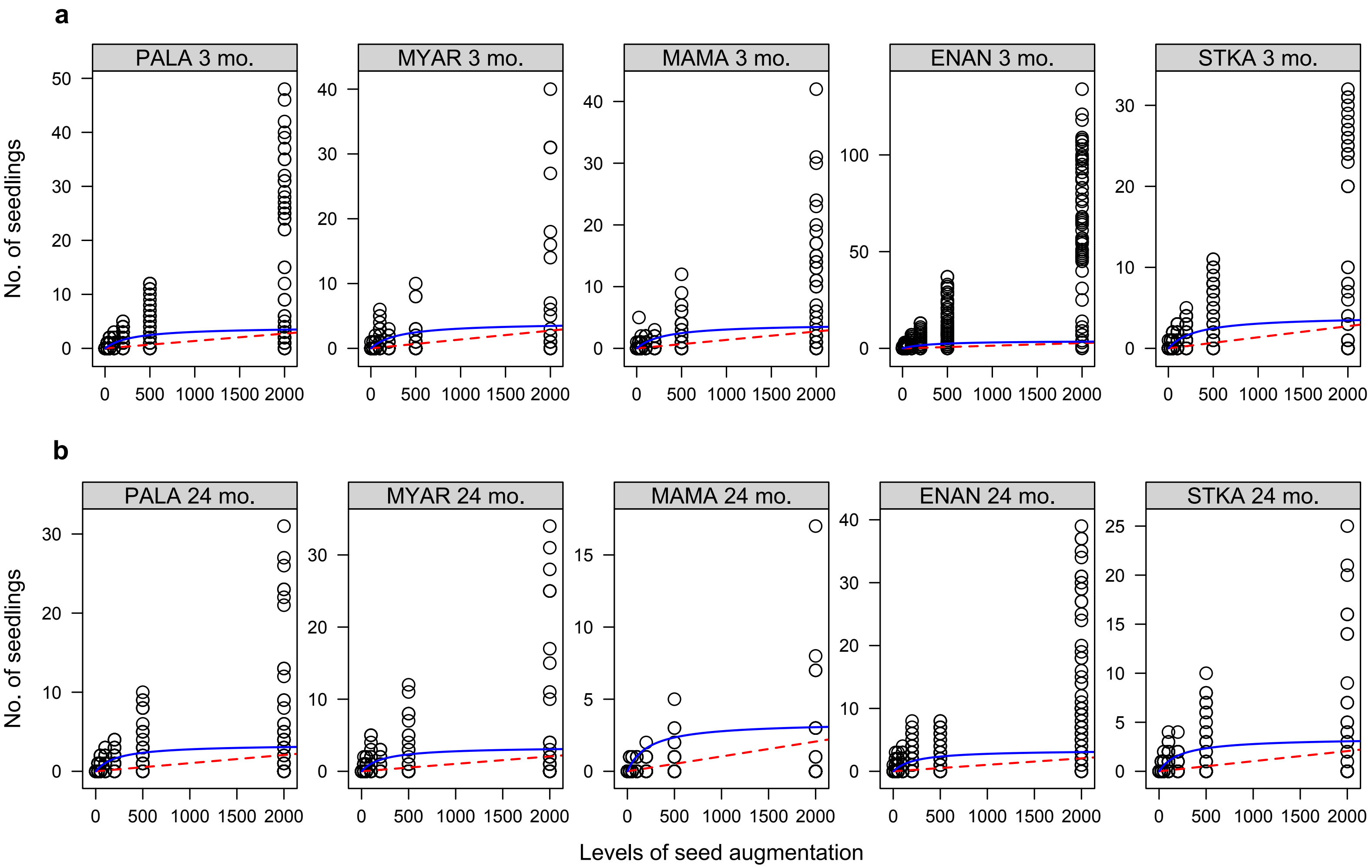

Figure S3. Fit of the recruitment functions to species-specific seed augmentation data. Each of the panels depicts the recruitment function of a species at either (a) three months or (b) 24 months after sowing, with species including: *Pancovia laurentii* (Pala), *Staudtia kamerunensis* (Stka), *Manilkara mabokeensis* (Mama), *Myrianthus arboreus* (Myar), and *Entandophragma utile* (Enut). The dashed line represents the *density-independent* (DI) model (fitting P_0 and S_{amb}) and the solid line represents the *density-dependent* (DD) model (fitting P_0, S_{amb}, and R_{max}). The level of seed augmentation is a multiple of ambient densities observed in nature for each species during the first year of this project. For all species, the full Beverton-Holt model (DD) provided an improved fit to the linear model (Supplementary Table 1).
