# Supplementary material for: Roles of Seed and Establishment Limitation in Determining Patterns of Afrotropical Tree Recruitment
Source: PLoS One. 2013 May 14;8(5):e63330. doi: 10.1371/journal.pone.0063330 (PMC3653939; doi:10.1371/journal.pone.0063330)
Supplement: Figure S4 — Results of limitation analysis for five tropical tree species, Entandophragma utile (Enut), Manilkara mabokeensis (Mama), Myrianthus arboreus (Myar), Pancovia laurentii (Pala), and Staudtia kamerunensis (Stka), at (a) three months and (b) 24 months after seed augmentation. Lines are establishment limitation (blue), seed limitation (black), density-dependent mortality (black), and density-independent mortality (green). For all species, establishment limitation becomes a stronger source of recruitment limitation than seed limitation () at very low seed input levels (4–6 times mean ambient seed densities). The strength of seed limitation declines sharply at seed addition levels below 1 seed m−2 (0.16–0.98 seeds m−2). For all species, density-independent mechanisms of seedling mortality more strongly prevent seedlings from achieving maximum population densities than density-dependent mechanisms until seed availability reaches high addition levels (, 222–765 times mean ambient seed rain densities). For four of five species, the point at which density-dependence more strongly limits seedling recruitment than either seed limitation or density-independent factors occurs at seed densities within the range observed in seed trap. (PDF) [file pone.0063330.s004.pdf]

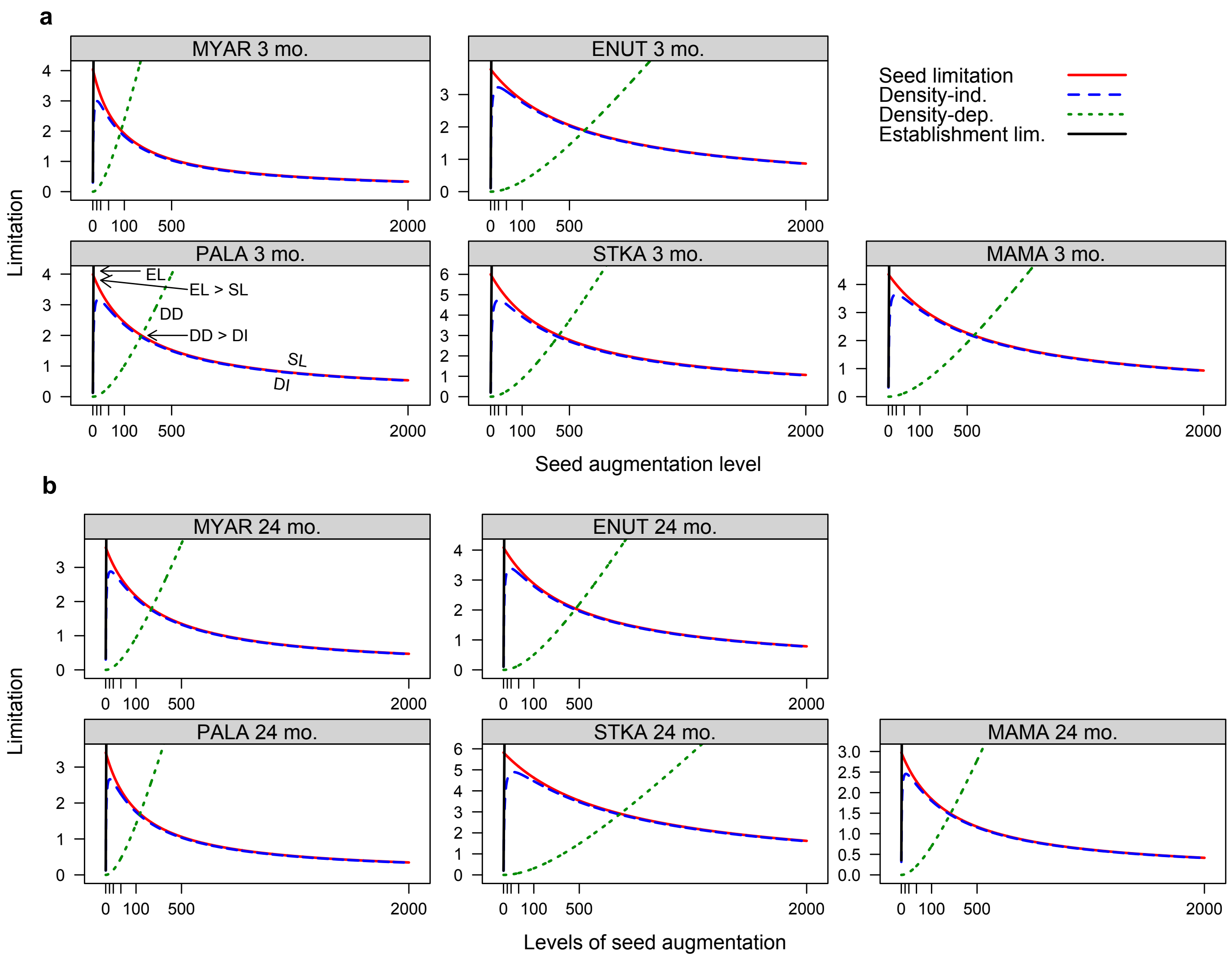

Figure S5. Results of limitation analysis for five tropical tree species, *Entandophragma utile* (Enut), *Manilkara mabokeensis* (Mama), *Myrianthus arboreus* (Myar), *Pancovia laurentii* (Pala), and *Staudtia kamerunensis* (Stka), at (a) three months and (b) 24 months after seed augmentation. Lines are establishment limitation (blue), seed limitation (black), density-dependent mortality (black), and density-independent mortality (green). For all species, establishment limitation becomes a stronger source of recruitment limitation than seed limitation ($EL > SL$) at very low seed input levels (4-6 times mean ambient seed densities). The strength of seed limitation declines sharply at seed addition levels below 1 seed m^{-2} (0.16-0.98 seeds m^{-2}). For all species, density-independent mechanisms of seedling mortality more strongly prevent seedlings from achieving maximum population densities than density-dependent mechanisms until seed availability reaches high addition levels ($DD > DI$, 222-765 times mean ambient seed rain densities). For four of five species, the point at which density-dependence more strongly limits seedling recruitment than either seed limitation or density-independent factors occurs at seed densities within the range observed in seed trap.
