# Supplementary material for: Roles of Seed and Establishment Limitation in Determining Patterns of Afrotropical Tree Recruitment
Source: PLoS One. 2013 May 14;8(5):e63330. doi: 10.1371/journal.pone.0063330 (PMC3653939; doi:10.1371/journal.pone.0063330)
Supplement: Table S1 — Parameter values from the density-independent (DI) model and density-dependent (DD) models for pooled and individual species. The DI model includes parameters for density-independent mortality, P 0, overdispersion, , and the random effect of Plot. The DD model includes a parameter for the maximum number of seedlings, R max. The CIs for each parameter are 95% credible intervals. When parameter results of DD model between three months (3 mo.) and 24 months (24 mo.) are compared, the DI parameter usually differs from 0 at three months, but not after 24 months, suggesting that the effect of seed addition begins to disappear by 2 years. Species codes are Pancovia laurentii (Pala), Staudtia kamerunensis (Stka), Manilkara mabokeensis (Mama), Myrianthus arboreus (Myar), and Entandophragma utile (Enut). Bolded AIC values highlight the best model for each Species and time combination, based on a difference of at least four. (DOCX) [file pone.0063330.s005.docx]

Table S1. Parameter values from the density-independent (DI) model and density-dependent (DD) models for pooled and individual species. The DI model includes parameters for density-independent mortality, *P*_0_, overdispersion, $k$, and the random effect of *Plot*. The DD model includes a parameter for the maximum number of seedlings, *R*_max_. The CIs for each parameter are 95% credible intervals. When parameter results of DD model between three months (3 mo.) and 24 months (24 mo.) are compared, the DI parameter usually differs from 0 at three months, but not after 24 months, suggesting that the effect of seed addition begins to disappear by 2 years. Species codes are *Pancovia laurentii* (Pala)*, Staudtia kamerunensis* (Stka)*, Manilkara mabokeensis* (Mama)*, Myrianthus arboreus* (Myar)*, and Entandophragma utile* (Enut). Bolded AIC values highlight the best model for each Species and time combination, based on a difference of at least four.

| Species | Time  mo. | Model | *P_0_* | *P_0_*  95% CIs | *k* | *k*  *95% CIs* | *R_max_* | *R_max_*  95% CIs | *Plot* | *Plot*  95% CIs | -2LL | AIC |
| --- | --- | --- | --- | --- | --- | --- | --- | --- | --- | --- | --- | --- |
| Pooled | 3 | DI | 0.002 | 0.002, 0.002 | 3.205 | 2.867, 3.543 | . | . | 0.319 | 0.068, 0.569 | 6793.8 | 6799.8 |
|  |  | DD | 0.021 | 0.014, 0.028 | 2.549 | 2.273, 2.826 | 5.101 | 4.791, 5.410 | 3.508 | 0.898, 6.117 | **6573.5** | **6581.5** |
| Pooled | 24 | DI | 0.001 | 0.001, 0.002 | 4.093 | 3.477, 4.709 | . | . | 1.984 | 0.593, 3.374 | 4218.9 | 4224.9 |
|  |  | DD | 0.019 | 0.009, 0.030 | 3.448 | 2.920, 3.976 | 4.325 | 3.926, 4.724 | 7.009 | 1.802, 12.216 | **4120.7** | **4128.7** |
| Pala | 3 | DI | 0.001 | 0.001, 0.002 | 1.436 | 1.061, 1.811 | . | . | 0.182 | . | 1443.5 | 1449.5 |
|  |  | DD | 0.013 | 0.005, 0.021 | 0.981 | 0.704, 1.257 | 3.993 | 3.478, 4.507 | 1.794 | 0.061, 3.527 | **1398.3** | **1406.3** |
| Pala | 24 | DI | 0.001 | 0.001, 0.001 | 2.714 | 1.809, 3.619 | . | . | 0.664 | . | 1030.1 | 1036.1 |
|  |  | DD | 0.015 | 0.000, 0.03 | 1.931 | 1.239, 2.623 | 3.405 | 2.644, 4.166 | 3.728 | -0.092, 7.547 | **1011.3** | **1019.3** |
| Stka | 3 | DI | 0.001 | 0.001, 0.002 | 4.149 | 2.203, 6.096 | . | . | 6.113 | . | 636.4 | 642.4 |
|  |  | DD | 0.006 | -0.004, 0.016 | 3.636 | 1.875, 5.396 | 3.779 | 2.21, 5.349 | 9.247 | 0.542, 17.952 | 632.7 | 640.7 |
| Stka | 24 | DI | 0.001 | 0.001, 0.002 | 2.252 | 1.187, 3.317 | . | . | 11.750 | . | 650.6 | 656.6 |
|  |  | DD | 0.009 | -0.001, 0.018 | 1.784 | 0.875, 2.692 | 4.085 | 2.989, 5.181 | 17.770 | 1.68, 33.862 | **641.6** | **649.6** |
| Mama | 3 | DI | 0.001 | 0.001, 0.002 | 2.022 | 1.228, 2.816 | . | . | 5.314 | . | 847.2 | 853.2 |
|  |  | DD | 0.008 | 0.001, 0.015 | 1.626 | 0.949, 2.303 | 4.363 | 3.395, 5.33 | 9.392 | 1.222, 17.562 | **833.2** | **841.2** |
| Mama | 24 | DI | 0.001 | 0.000, 0.001 | 3.662 | 1.083, 6.24 | . | . | 14.628 | . | 402.4 | 408.4 |
|  |  | DD | 0.009 | -0.004, 0.022 | 2.706 | 0.724, 4.689 | 2.977 | 1.698, 4.256 | 23.584 | -0.085, 47.254 | **395.7** | **403.7** |
| Myar | 3 | DI | 0.002 | 0.002, 0.002 | 1.046 | 0.811, 1.281 | . | . | 0.745 | . | 2014.9 | 2020.9 |
|  |  | DD | 0.014 | 0.010, 0.018 | 0.484 | 0.353, 0.616 | 6.002 | 5.583, 6.42 | 1.305 | 0.257, 2.354 | **1858.6** | **1866.6** |
| Myar | 24 | DI | 0.002 | 0.002, 0.002 | 1.652 | 1.135, 2.169 | . | . | 1.834 | . | 1206.7 | 1212.7 |
|  |  | DD | 0.008 | 0.003, 0.012 | 1.405 | 0.957, 1.853 | 5.823 | 4.625, 7.022 | 3.914 | 0.466, 7.362 | **1189.0** | **1197.0** |
| Enut | 3 | DI | 0.001 | 0.001, 0.001 | 1.102 | 0.737, 1.466 | . | . | 2.061 | . | 1256.1 | 1262.1 |
|  |  | DD | 0.023 | 0.008, 0.037 | 0.554 | 0.328, 0.779 | 4.051 | 3.534, 4.567 | 7.336 | 1.441, 13.231 | **1180.6** | **1188.6** |
| Enut | 24 | DI | 0.001 | 0.001, 0.002 | 2.261 | 1.246, 3.277 | . | . | 6.377 | . | 739.9 | 745.9 |
|  |  | DD | 0.012 | -0.001, 0.024 | 1.517 | 0.732, 2.301 | 3.584 | 2.765, 4.403 | 12.126 | 1.484, 22.769 | **724.1** | **732.1** |
